# Increasing compound events of extreme hot and dry days during growing seasons of wheat and maize in China

**DOI:** 10.1038/s41598-018-34215-y

**Published:** 2018-11-12

**Authors:** You Lu, Hongchang Hu, Chao Li, Fuqiang Tian

**Affiliations:** 1 Department of Hydraulic Engineering, State Key Laboratory of Hydroscience and Engineering, Tsinghua University, Beijing, 100084 P. R. China; 2 Pacific Climate Impact Consortium, University of Victoria, Victoria, British Columbia V8W 2Y2 Canada

## Abstract

Compound events of climate extremes such as extremely high temperature and low precipitation during crop growing seasons can greatly affect agricultural production and food security. No study has investigated how Compound Extreme Hot and Dry days (CEHD days) during crop-growing seasons have changed or will change in response to climate warming. Based on observations, we find upward trends in CEHD days during wheat and maize growing seasons in China in the historical period 1980–2015. These trends are remarkably different during wheat and maize growing seasons, pointing to the need for targeted analysis focusing on crop-specific growing seasons. Projections of future temperature and precipitation from the Coordinated Regional Climate Downscaling Experiment show that upward trends will continue into future. On average over China, the frequencies of CEHD days during wheat and maize growing seasons are projected to increase respectively by 168% and 162% in 2036–2050 relatively to 1980–2015 under the RCP8.5 emissions scenario. The projected increases may have serious implications for China’s food production, adding to the need for resilience planning to limit the impacts of growing-season CEHD days.

## Introduction

Food production is seriously affected by climate extremes^1,2^, which are becoming more frequent and more severe under climate change^3^, and food security will be consequently confronted with increasing risks^4^. China sustains 20% of the world’s population with only 8% of the world’s arable areas^5,6^, and agricultural economy of China is also the globally largest one, employing about 50% of the workforce in China^7,8^. Food security in China is therefore of critical importance, affecting not only the life of more than 1.3 billion people but also the international food market and global economy^9,10^. As such, changes in climate extremes have great implications to food security in China^11^, and require critical investigations, as has been done in many other regions of the world^12–15^.

Existing studies have evaluated changes in temperature and precipitation extremes in China based on both observations and climate model simulations^16,17^, and have found that the frequencies of heat waves in Eastern China and droughts in Northern China have increased over the last decades^18^ and will continue to increase with human-induced global warming. Precipitation and temperature extremes are evaluated separately in these studies^19^. However, as a compound weather/climate event (i.e., combination of multiple drivers and/or hazards contributing to societal or environmental risk)^20^, the concurrence of extreme high temperature and low precipitation can intensify the negative effect on the ecosystems and society, causing worse impacts on food production and security than the independent impact from each vairable^21–24^. For example, the 2014 California drought is severely aggravated by concurrent extreme heat waves^25^. Recent studies have investigated the impacts of compound events of heat waves and droughts on crop yields, and have found that these compound events can explain a significant portion of inter-annual production variability^26,27^. Considering only one variable at a time may therefore lead to underestimation of risks^28^. A growing body of research has focused on the impacts of global warming on compound extreme events^20^. For example, recent studies has reported that the frequency of months with high temperature and low precipitation is increasing over Europe based on a suite of regional climate model simulations^29^, as well as in other regions across the globe based on global climate model simulations from the Coupled Model Intercomparison Project Phase 5^30^. Compound extreme events in previous studies are calculated over a season or all year round^16,31^. Given the fact that only compound events of extreme temperature and precipitation during crop growing seasons will be felt by crops, and that growing seasons vary substantially among crops and regions, a more informative assessment of the evolution of compound events of extreme temperature and precipitation in response to global warming should refer to the growing seasons of particular crops in particular regions.

Here we study how the frequency of Compound Extreme Hot and Dry (CEHD) days during the growing seasons of wheat and maize in China has changed since 1980 based on daily temperature and precipitation observations from the China Meteorological Forcing Dataset. We then exploit an ensemble of regional climate model simulations from the Coordinated Regional Climate Downscaling Experiment (CORDEX)^32^ over East Asia (covering China) for the period 1980–2050 to investigate how the frequency of CEHD days is projected to change by mid-century under the Representative Concentration Pathway 8.5 emissions scenario.

## Data and Methodology

### Data

The timespan of growing seasons for rain-fed wheat and maize in China is retrieved from the MIRCA2000 dataset with a horizontal resolution of 5 arc-minute^33^. We focus this study on the rain-fed wheat and maize for a two-fold consideration: first, wheat and maize are two main crops in China accounting for 56.5% of the food production^34^, and second, the rain-fed crops are more prone to be affected by extreme hot and dry days compared to irrigated ones^27,35^. The length of a growing season may vary among crops and from place to place. According to the MIRCA2000 dataset, the growing season lengths are around 150 days for maize throughout China, while for wheat they vary from 120 days to 300 days among regions.

We acquire temperature and precipitation observations for 1980–2015 from the China Meteorological Forcing Dataset, which provides 3-hourly land surface meteorological variables with a spatial resolution of 0.1 degree over China. The 3-hourly temperature and precipitation observations are aggregated into daily values for individual grid cells. We also use daily temperature and precipitation from an ensemble of 5 climate simulations for the period 1980–2050 at a spatial resolution of 0.44 degree. These simulations are from 5 regional climate models participating in the CORDEX program over East Asia, with one simulation from each model. Identifiers of these 5 regional climate models are: HadGEM3-RA, RegCM4, SNU-MM5, SNU-WRF and YSU-RSM. Simulations from these regional models are driven by the same simulation from the global climate model HadGEM2-AO, which uses “all” forcing for the historical period ending in 2005 (solar and volcanic forcing, greenhouse gases, aerosols, ozone and land use) and follows the RCP8.5 emissions scenario for 2005 onward. The SNU-MM5 simulations for the period 2036–2050 are not achieved, and thus are not included in our analysis. See Table 1 for details of the used datasets. In order to keep consistent with the spatial resolution of the MIRCA2000 dataset, temperature and precipitation observations and simulations are linearly interpolated to 5-arc-minute.

**Table 1:** Basic information of the meteorological data. All of the RCM data can be downloaded from http://www.cordex.org/.

Data	Source	Study Period	Spatial and Temporal Resolution
China Meteorological Forcing Dataset (Observed Data)	Chen *et al*.^33^	1980–2015	0.1° and 3-hour
RCM Data from CORDEX Driven by GCM HadGEM2-AO (Simulated Data)	HadGEM3-RA	1980–2005 under Historical;	0.44° and daily
		2006–2050 under RCP4.5 and RCP8.5	
	RegCM4	1980–2005 under Historical;	0.44° and daily
		2006–2050 under RCP4.5 and RCP8.5	
	SNU-MM5	1980–2005 under Historical;	0.44° and daily
		2006–2050 under RCP4.5 and 2006–2035 under RCP8.5	
	SNU-WRF	1980–2005 under Historical;	0.44° and daily
		2006–2049 under RCP4.5 and RCP8.5	
	YSU-RSM	1980–2005 under Historical;	0.44° and daily
		2006–2050 under RCP4.5 and RCP8.5	

For both observed data and RCM data, precipitation data and temperature data are used in this study and all meteorological data are interpolated to daily data with spatial resolution of 5 arc-minute to be consistent with the crop data.

### Methodology

We define a day within the growing season of a given crop (wheat or maize) as a CEHD day if temperature and precipitation of that day are respectively above and below certain extremely high temperature and low precipitation thresholds. To define extreme hot days, we use dynamic extreme high temperature thresholds, with one threshold for each calendar day, which is calculated as the 90^th^ percentile of the distribution of daily mean temperatures aggregated across a time window of 21 days centered on that calendar day and across all years in the baseline period 1980–2005. The use of dynamic temperature thresholds accounts for the varying optimum growth temperatures and thermal tolerance of crops in different growth stages^36^ as well as the large seasonality and spatial variability of temperatures in China. Unlike for temperature, the 10^th^ percentile of precipitation amounts of all growing-season wet days (with precipitation of non-zero value) during 1980–2005 is taken as the extreme low precipitation threshold. We excluded dry days when calculating the precipitation percentile since in some areas the majority of days during both wheat and maize growing seasons are dry. Examination of the studied daily precipitation observations and simulations reveals no significant trend in the frequency of growing-season wet days in most wheat and maize-cropped regions in China. This means that long-term variations in the wet-day frequency if any will not qualitatively affect our major conclusions.

In this study, we are particularly interested in the frequency of CEHD days during the growing seasons of rain-fed wheat and maize. Although this frequency of CEHD days does not resolve the persistence properties of hot and/or dry spells, which are similarly key for the impacts of droughts and heat waves to crop yields, it reflects the overall conditions of extremely severe weather and climate conditions (i.e., low water availability and high heat stress) for a rain-fed crop during its entire growing season. In this sense, the frequency of CEHD days still represents an important indicator for potential impacts of global warming on agriculture and food security. We find that the defined growing-season frequency of CEHD days is substantially different from the frequency of CEHD days in a season or all year around, as well as between wheat and maize (Fig. S1), indicating the need for targeted analysis focusing on crop-specific growing seasons. It is realized that some growth stages (e.g., the emergence and anthesis stage of wheat) are more sensitive to severe weather and climate conditions than others^15^. Ideally, investigating changes in the frequency of CEHD days during some critical growth stages is desired. This is not addressed in the present study due to the lack of reliable information for the dates of those critical growth stages.

We estimate trend in the frequency growing-season CEHD days using the nonparametric Sen’s slope estimator^37,38^. We use the Mann-Kendall test at the 5% significance level to determine whether the estimated trend is statistically significantly different from zero^39^. Trend estimation is implemented on the individual time series of the growing-season CEHD-day frequency at each grid cell, and on the averaged time series over all cropped grid cells weighted by crop cultivated areas (for each studied crop). Percentage change is expressed as the ratio (in percent) of the total trend (i.e., Sen’s slope multiply by the number of years) during a given period (e.g., 1980–2015) divided by the 1980–2005 baseline climatology, and thus can be interpreted as the change in the CEHD-day frequency during that given period relative to the 1980–2005 climatology.

## Results and Discussion

**Figure 1:**
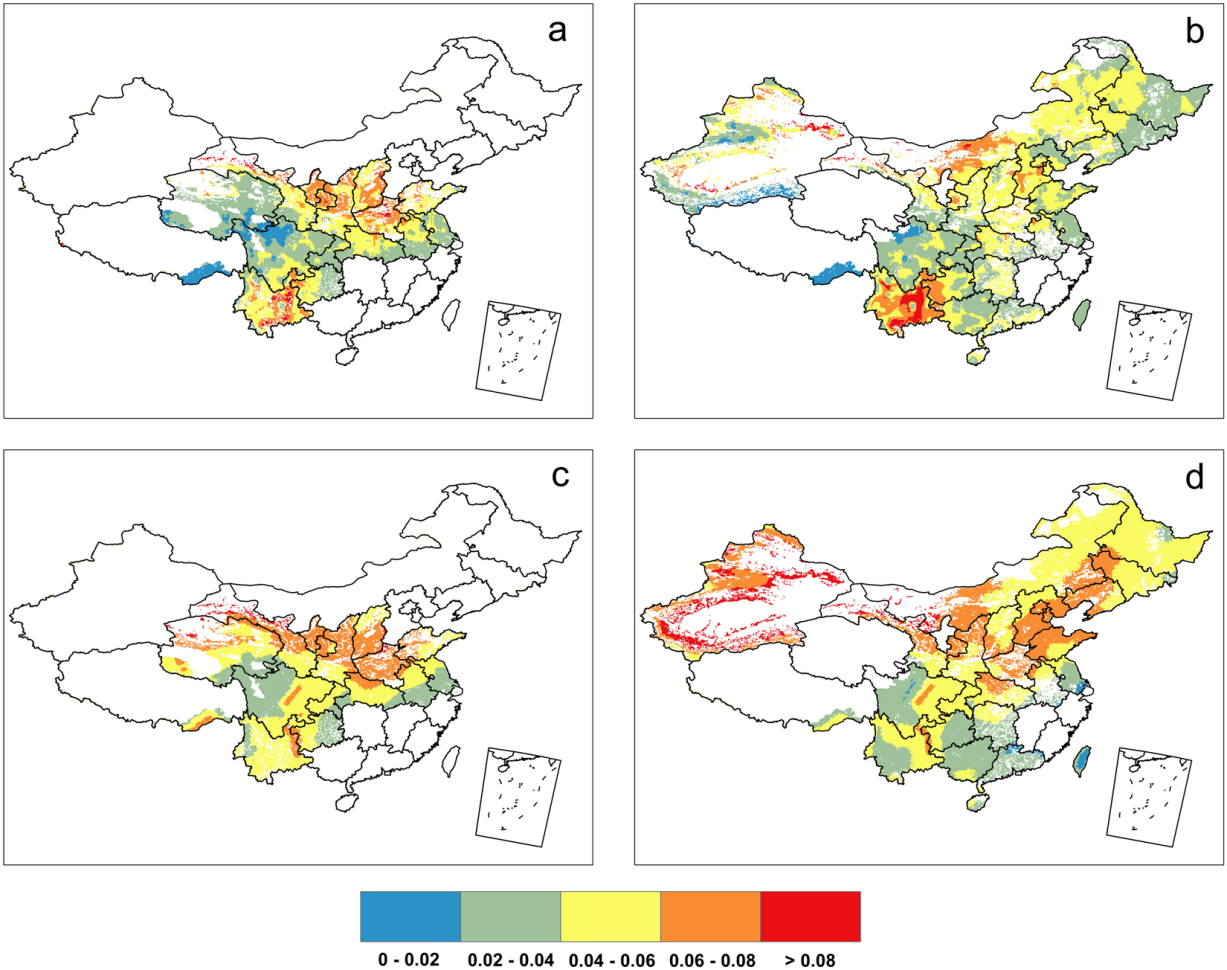
Climatology of the mean of CEHD-day frequency in growing season from observations over 1980–2015 for wheat and maize (**a,b**) and multi-model mean of the frequency from simulations over 1980–2015 for wheat and maize (**c,d**). (All of the items were generated with ArcGIS 9.3, https://www.arcgis.com/features/index.html).

Figure 1 shows the 1980–2015 climatological mean growing-season CEHD-day frequency for rain-fed wheat and maize in observations (a-b) and the ensemble mean of multi-model simulations (c-d). For both wheat and maize, the climatological CEHD-day frequency exhibits remarkable spatial variations, with some noticeable hotspots exhibiting extremely high stress from CEHD days (Fig. 1a,b). In general, wheat and maize in northern China are suffering more CEHD days during their growing seasons than those in southern China, with the exception of the Southwest China (east of the Tibetan Plateau), where CEHD-day frequencies can be as high as 10%, particularly for maize. Other hotspots of high climatological CEHD-day frequency occur to wheat in North China Plain, and to maize in some localized regions in North China Plain and Northwest China. North China Plain is China’s major wheat production region. The high climatological stress from growing-season CEHD days for wheat in this region highlights the relatively high vulnerability of China’s wheat production in a sense that a small increase in the proportion of growing-season CEHD days may cause large production loss.

There are also obvious differences in the frequency of CEHD days faced by wheat and maize. For example, wheat in North China Plain are experiencing more frequent CEHD days than maize, while an opposite tendency is observed in Southwest China. Such a contrast between the CEHD-day frequency of wheat and maize suggests the importance of considering compound negative weather and climate extremes during crop-specific growing seasons when assessing the potential impacts of climate change on agriculture and food security.

In general, the ensemble mean of multi-model simulations of 1980–2015 (under historical scenario for 1980–2005 and under RCP4.5 scenario for 2006–2015) broadly reproduce the overall spatial patterns of the observed 1980–2015 growing-season CEHD-day frequency for both wheat and maize (Fig. 1c,d). Noticeably, the observed pattern of more CEHD days in northern than southern China also appears in climate model simulations. As expected, the spatial distribution of the multi-model ensemble mean CEHD-day frequency is smoother than that observed as it is less affected by internal climate variability. At local to regional spatial scales, biases are obvious in the ensemble mean of the modeled CEHD-day frequency. For example, the CEHD days are simulated to occur more frequently during wheat growing season in Southwest China and during maize growing season in Northwest China and part of North China plain, while they are simulated to occur less frequently during maize growing season in part of Southwest China. Compared to wheat, the biases for maize are more pronounced. Despite these biases, the observed 1980–2015 climatological mean growing-season CEHD-day frequency lies within the range of individual simulations over 95% of the rain-fed wheat growing areas and over 96% of the rain-fed maize growing areas in China, though the spreads of the ensembles are relatively large. We also exploited the climatology of the median of frequency from observations and simulations over 1980–2015 (Fig. S2), which shows similar results with the climatology of the mean.

**Figure 2:**
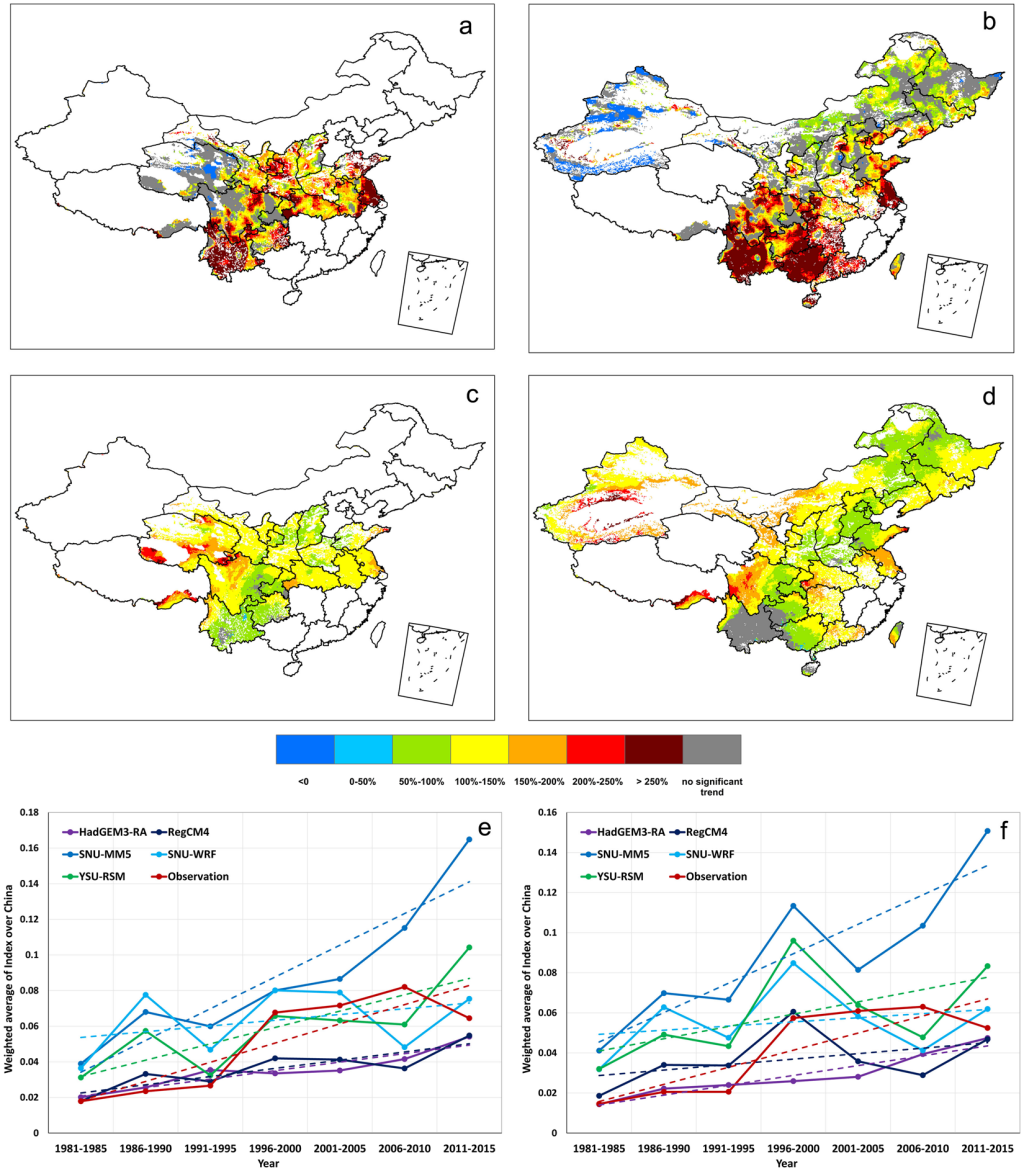
Observed percentage change during 1980–2015 relative to the mean of the frequency of 1980–2015 for wheat and maize (**a,b**), multi-model mean percentage change during 1980–2015 relative to the mean of the frequency of 1980–2015 for wheat and maize (**c,d**), and non-overlapping 5-year mean of the 1981–2015 weighted average frequency time series based on observations and each individual model simulation for wheat and maize (**e,f**). ((**a**–**d**) were generated with ArcGIS 9.3, https://www.arcgis.com/features/index.html, and (**e**,**f)** were generated with Microsoft Excel 2013, https://products.office.com/en-us/excel).

The observed 1980–2015 trends in the frequency of CEHD days during rain-fed wheat and maize growing seasons are shown in Fig. 2a,b. The CEHD days have become more frequent since 1980 over the majority of the growing areas for both wheat and maize. In particular, the upward trends are statistically significant at the 5% level in over 70% and 63% of the wheat and maize growing areas, respectively. Over most of the regions where the upward trends are significant, increases in the frequency of CEHD days during 1980–2015 can be as high as >250% of the local climatological means during the period of 1980–2005. We note that the most pronounced increases occurs in Southwest China for both wheat and maize, and in North China Plain for wheat. These regions are also where high climatological stress from CEHD days already exists (Fig. 1a,b), suggesting that rain-fed wheat and maize in these regions are at a high level of vulnerability to global warming-induced severe conditions of low water availability and high heat stress. We also notice a decreasing tendency in the frequency of CEHD days, but only in very small areas in Northwest China. The robustness of trends can be proved in 93% of the wheat grids and 88% of the maize grids if any one year data were to be removed.

Broadly consistent with observations, significant increases in the frequency of growing-season CEHD days are also found almost everywhere of the growing areas for both wheat and maize based on the multi-model simulated ensemble mean trend during 1980–2015, except for Southwest China where climate models fail to show significant change in CEHD days during the growing season of maize. Despite the overall agreement in the widespread intensification of growing season CEHD days between observations and simulations, we also see substantial discrepancies however, noticeably in the magnitude of change. For example, there are regions where the frequency of CEHD days does not show significant change in observations, but are simulated to increase significantly. These regions include parts of the wheat growing areas in Northwest China and parts of maize growing areas in northern China. On the other hand, over regions where significant increases have been observed, climate models greatly underestimate their magnitudes. After averaging over all growing areas, we find that the observed average trends lie within the range of those simulated by individual climate models (partly because the influence of internal climate variability is reduced by spatial averaging), though close to the lower boundary of the range (Fig. 2e,f). In summary, although climate simulations are biased in local to regional changes in the growing-season frequency of CEHD days, they reproduce reasonably well the upward trends averaged over the growing areas of rain-fed wheat and maize in China.

**Figure 3:**
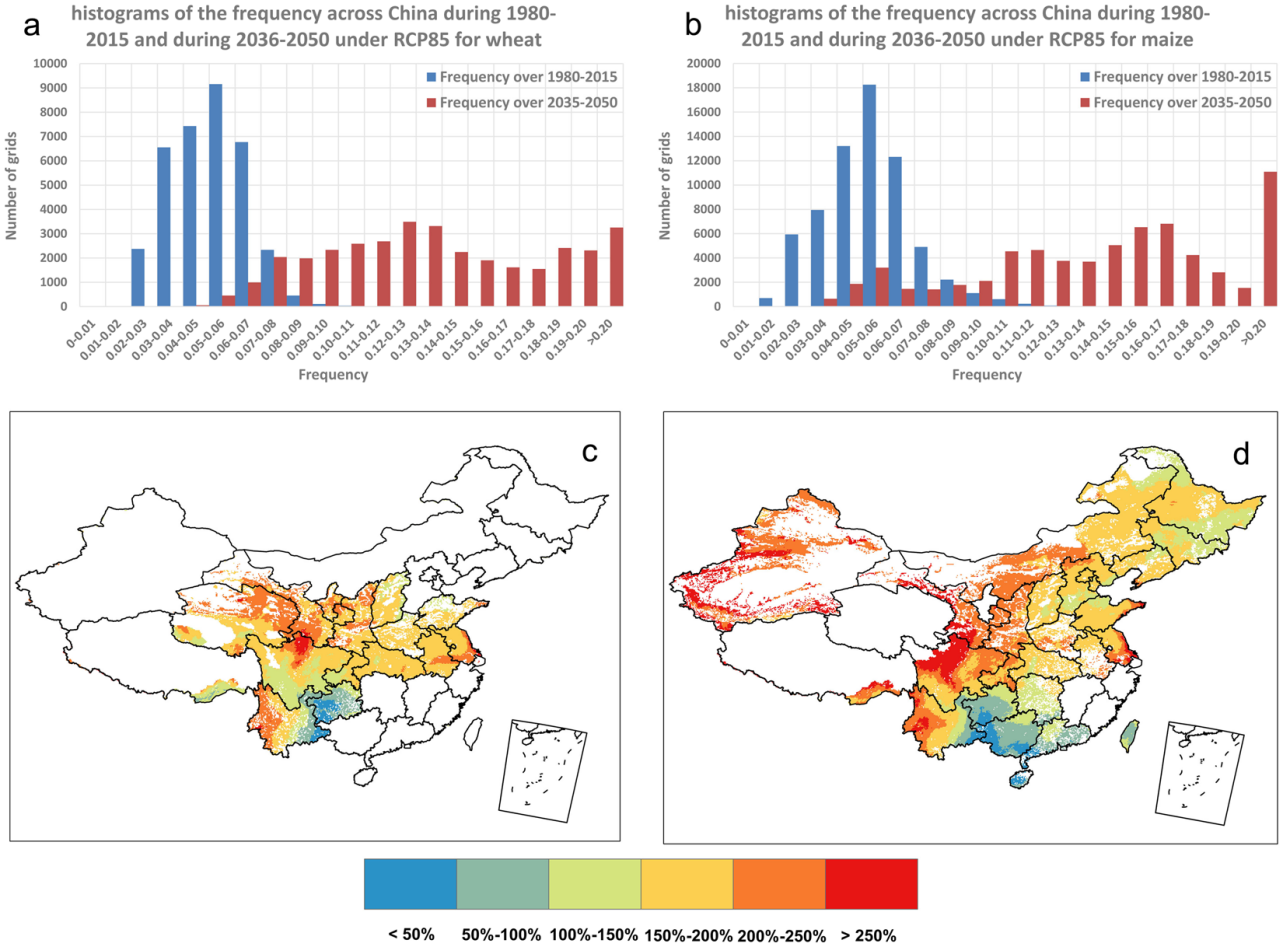
Histograms of the frequency across China during 1980–2015 and during 2036–2050 under RCP8.5 for wheat and for maize (**a,b**) and maps of the relative change of frequency during 2036–2050 under RCP8.5 relative to frequency during 1980–2015 (**c**,**d**). ((**a**,**b)** were generated with Microsoft Excel 2013, https://products.office.com/en-us/excel, and (**c**,**d**) were generated with ArcGIS 9.3, https://www.arcgis.com/features/index.html).

Looking to future, the frequency of growing-season CEHD days is projected to further increase under the RCP8.5 emissions scenario. Histograms in Fig. 3a,b compares the climate model simulated growing-season frequency of CEHD days for wheat (Fig. 3a) and maize (Fig. 3b) in a near future period 2036–2050 under RCP8.5 relative to the historical period 1980–2015. To plot the histograms, we calculated the mean of CEHD-day frequencies during a given period (e.g., 1980–2015 or 2036–2050) from all crop-growing grid cells, in the hope of reducing the influence of internal climate variability^40^. Apparently, the histograms of the aggregated growing-season frequency of CEHD days will shift to higher levels for both wheat and maize as climate warms with larger variance, resulting in more CEHD days in the course of the growing season. It is estimated that the averaged frequency weighted by crop areas will increase by as large as 168% for wheat and 162% for maize in 2035–2050 compared to 1980–2015 over China. A detailed examination of the histogram shifts reveals that the median of the CEHD-day frequency distribution for wheat in 2036–2050 will exceed the very end of the upper tail of the distribution in the historical period (Fig. 3a), implying that in some wheat-growing regions severe weather conditions of low water availability and high heat stress which have occurred very rarely in the historical period 1980–2015 will become the norm in 2036–2050. With larger variability, the frequency of growing-season CEHD days for maize across regions also shows shift to higher levels, and the median of the future 2036–2050 distribution is projected to exceed the historical maximum (Fig. 3b). Further, the upper tail of the CEHD-day frequency distribution in the future period extends far away from the record value experienced during the historical period, implying unprecedented severe dry and heat conditions are likely to occur during growing seasons of wheat and/or maize in some regions in near future. These projected increases, if occurred, would cause serious impacts to China’s food production and security, thus point to the pressing need for resilience planning to limit the impacts of the potential increases of crop growing-season CEHD days. Under RCP4.5 emissions scenario, similar shifts in the aggregated distribution of the growing-season frequency of CEHD days are projected to occur for both wheat and maize, but with considerably smaller magnitudes (Fig. S3), suggesting the benefits from reducing emissions of greenhouse gases. These results are consistent with Sun, *et al*.^41^, where concurrent droughts and heat waves are projected to occur more often.

The spatial distributions of the projected changes in the climatological mean of the growing-season CEHD-day frequency in 2036–2050 under RCP8.5 relative to 1980–2015 are presented in Fig. 3c,d. It is seen that the frequency of growing-season CEHD days is projected to increase more in the North than in the South for both wheat and maize, with remarkable swathes of great increases over 250% relative to 1980–2015 occurring in northwestern China. Considering the fact that in general wheat and maize in northern China are experiencing more climatological CEHD days during their growing seasons than those in southern China (Fig. 1a,d), the projected larger increases in CEHD days in northern China suggest the potentially high vulnerability of wheat and maize in this region to further climate warming.

We note that the projected increases of growing-season CEHD days at small scales can be largely uncertain (i.e., values mapped in Fig. 3c,d), due mainly to climate model deficiency in simulating temperature and precipitation at small scales (grid cell in space and daily in time) and the large influence of internal climate variability at these scales. Joint bias correction that accounts for interaction between temperature and precipitation is expected to partly address this issue^39^. Nevertheless, joint bias correction requires very large samples of observations as reference to obtain a reliable correction function, and may affect the model simulated trend^37^. Whether or not the simulated trend should be preserved is still in debate^38^. We therefore base our analyses of future CEHD days on native model projections, and focus on changes in the spatially aggregated CEHD days over all growing areas across China (i.e., results shown in Fig. 3a,b). The overall agreement between model simulations and observations in the spatially aggregated 1980–2015 trends indicates that the projected overall increases in growing-season CEHD days for wheat and maize should be informative for relevant adaptation planning.

## Summary and Conclusion

Concurrent climate extremes have much more severe impact on food security compared to extreme hot and dry days individually, especially when occurring in crop growing seasons. We quantify the frequencies of CEHD days during different crop growing seasons across China in response to climate warming for better understanding of the impacts on food security. During 1980–2005, North China Plain, Northwest China and part of Southwest China suffered more frequent CEHD days than the remaining areas, while significant upward trends of frequencies can be observed in most areas across China for both wheat and maize. There are also obvious differences in the frequency and trend of CEHD days faced by wheat and maize, indicating the importance of focusing on different crop growing seasons. Simulations can broadly reproduce the observed frequencies and trends over China for wheat and maize, and we find that the observed average trends weighted by cultivated areas lie within the range of those simulated by individual climate models, although at local scale the simulated frequencies and trends can be biased. In the future, the upward trends are projected to continue under RCP8.5 scenario, and Northern China is projected to bear more increase of CEHD days in growing seasons than Southern China, suggesting the potentially high vulnerability of wheat and maize in this region to further climate warming.

In future studies, the persistent periods of dry conditions should be considered to define a drought event and analyze the severity of concurrent climate extremes. Analysis focusing on different growth stages based on detailed crop information, particularly focusing on the stage when crops are more sensitive to concurrent climate extremes, will provide more accurate and useful implications. Additionally, the role of human activities on the reported observations should be discussed^41^, uncertainties from different data source need to be further discussed, and the relationships between the CEHD-day frequencies and crop production reduction need more investigation.

## Electronic supplementary material

Supplementary Information
