# Supplementary material for: Increasing compound events of extreme hot and dry days during growing seasons of wheat and maize in China
Source: Sci Rep. 2018 Nov 12;8:16700. doi: 10.1038/s41598-018-34215-y (PMC6232161; doi:10.1038/s41598-018-34215-y)
Supplement: Supplementary file 1 — Supplementary Information [file 41598_2018_34215_MOESM1_ESM.docx]

Supplementary Information

**Increasing compound events of extreme hot and dry days during growing seasons of wheat and maize in China**

Authors: You Lu^1^, Hongchang Hu^1^*, Chao Li^2^, Fuqiang Tian^1^

1. Department of Hydraulic Engineering, State Key Laboratory of Hydroscience and Engineering, Tsinghua University, Beijing 100084, P. R. China
2. Pacific Climate Impact Consortium, University of Victoria, Victoria, British Columbia, V8W 2Y2, Canada

*Corresponding author information:

Email: huhongchang@tsinghua.edu.cn

Tele: +86 010 6277 3396

Fax: +86 010 6279 6971


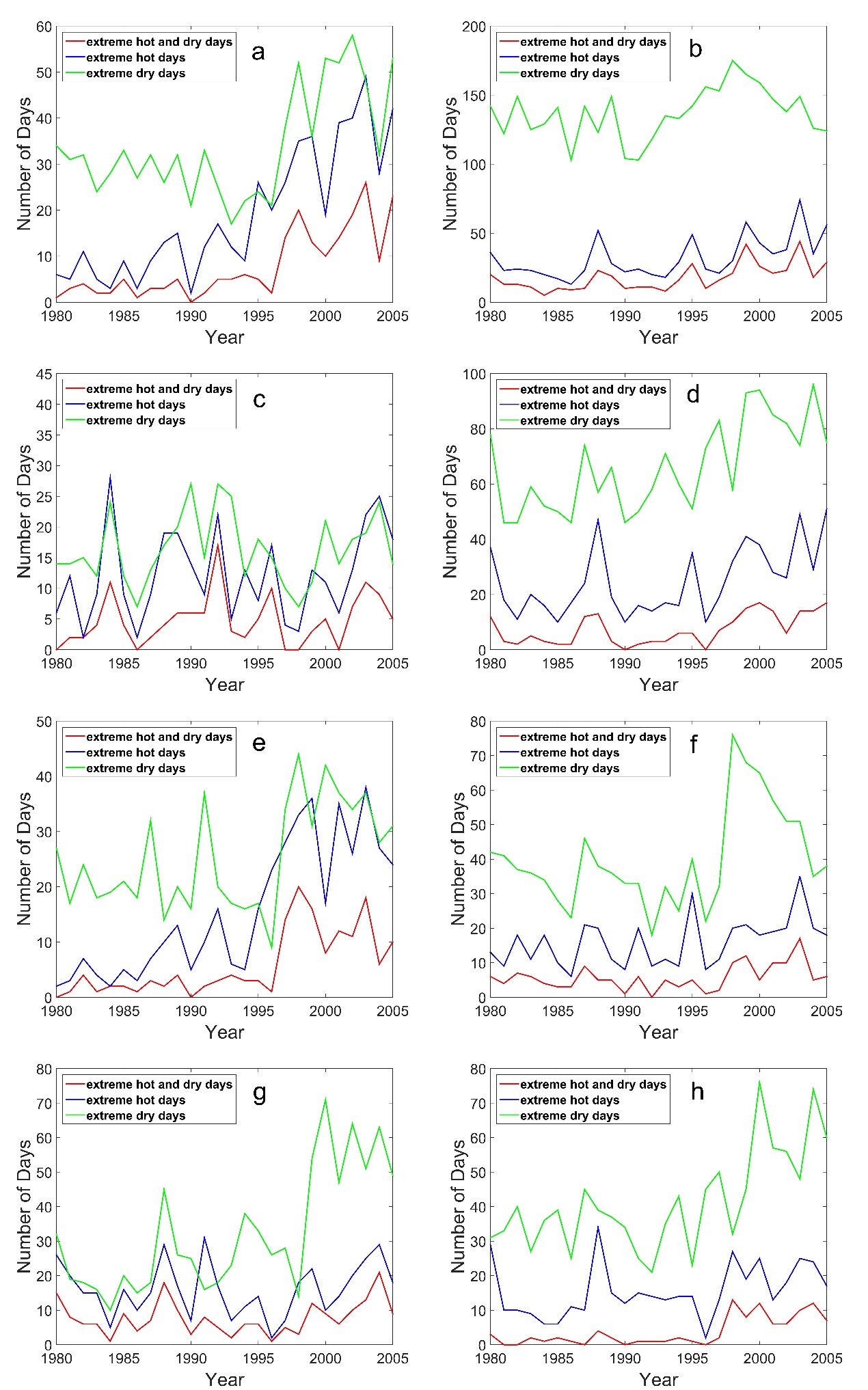


Supplementary Figure S1. The total number of concurrent extreme hot and dry days, extreme hot days and extreme dry days over growing seasons of wheat (a-d) and maize (e-h) in different areas, including (105^o^E, 35^o^N) in Northwest China (a,e), (115^o^E, 35^o^N) in North China Plain (b,f), (105^o^E, 25^o^N) in Southwest China (c,g), (115^o^E, 30^o^N) in Southeast China (d) and (113^o^E, 25^o^N) in Southeast China (h). The total numbers are calculated based on observed meteorological data from 1980 to 2005. (All of the items were generated with Matlab R2015a, https://www.mathworks.com/products/matlab.html)


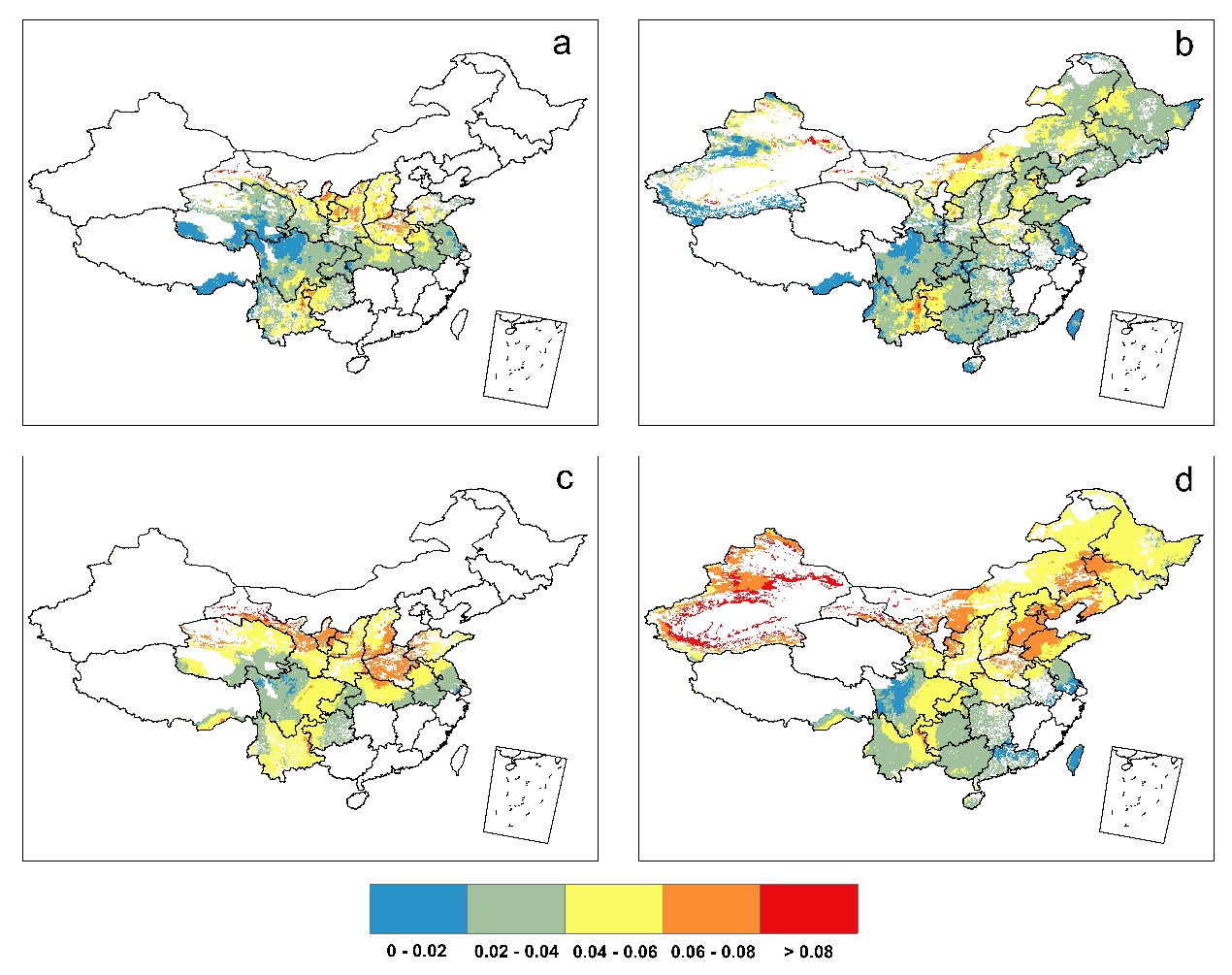


Supplementary Figure S2. Median of CEHD-day frequency in growing season from observations over 1980-2015 for wheat and maize (a,b) and multi-model median of the frequency from simulations over 1980-2015 for wheat and maize (c,d). (All of the items were generated with ArcGIS 9.3, https://www.arcgis.com/features/index.html).


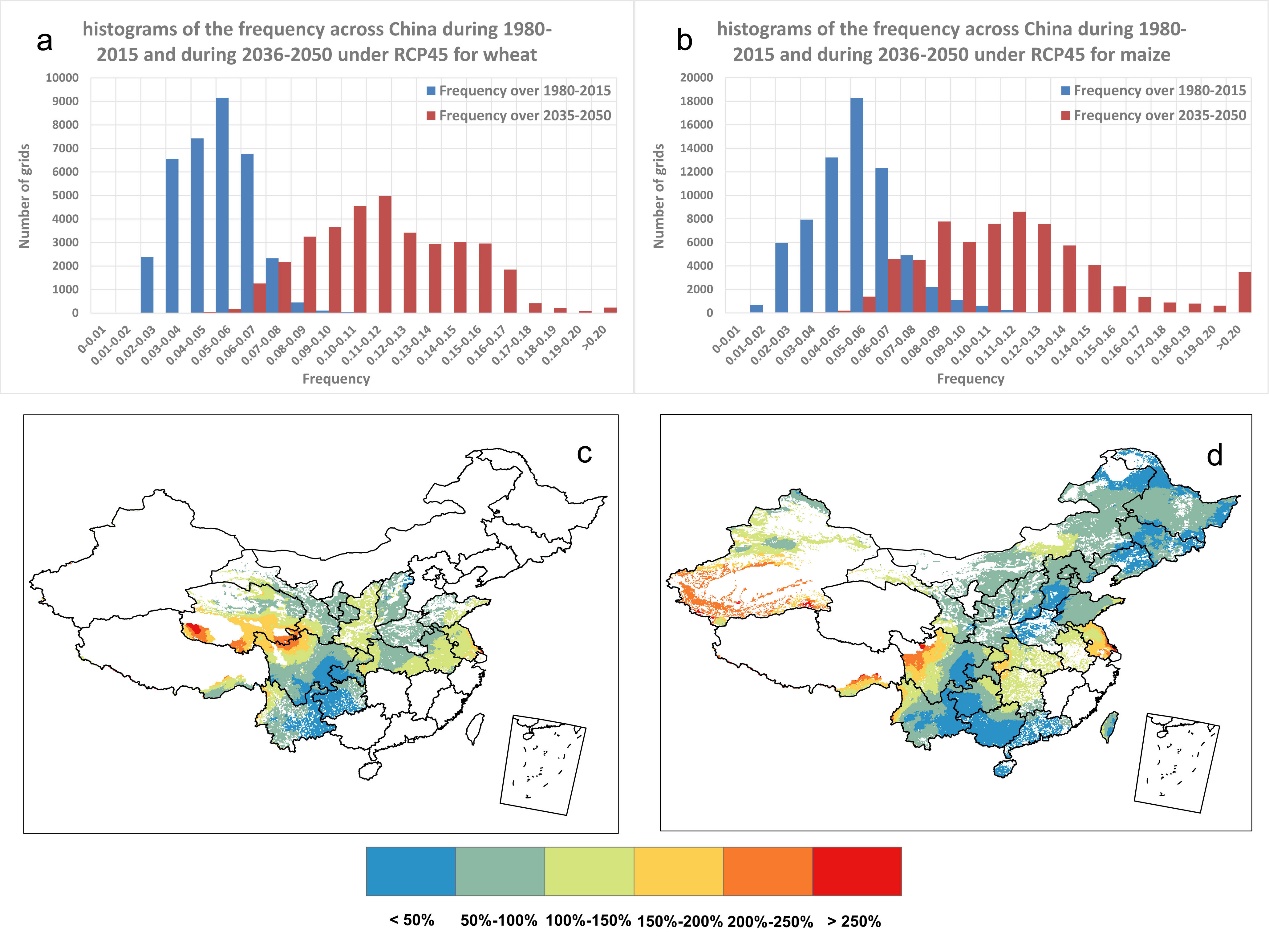


Supplementary Figure S3. Histograms of the frequency across China during 1980-2015 and during 2036-2050 under RCP4.5 for wheat and for maize (a,b) and maps of the relative change of frequency during 2036-2050 under RCP4.5 relative to frequency during 1980-2015 (c,d) (Figure 3a-3b were generated with Microsoft Excel 2013, https://products.office.com/en-us/excel, and Figure 3c-3d were generated with ArcGIS 9.3, https://www.arcgis.com/features/index.html).
